# Prevalence, demographics, comorbidities, and treatment patterns of patients with the trigeminal autonomic cephalalgias: a retrospective analysis of United States electronic health records

**DOI:** 10.1186/s12883-025-04314-1

**Published:** 2025-07-21

**Authors:** Leon S. Moskatel, Oyindamola Ogunlaja, Niushen Zhang

**Affiliations:** Department of Neurology, Stanford University, 211 Quarry Road, Suite #206, Palo Alto, CA 94304 United States of America

**Keywords:** TAC, Hemicrania continua, Cluster headache, Paroxysmal hemicrania, SUNCT, Epidemiology

## Abstract

**Background:**

The study of the trigeminal autonomic cephalalgias (TAC) has been limited by difficulty aggregating sufficient numbers of patients. We used the Epic Cosmos electronic health record research platform to harness nationwide data from health care systems across the United States using the Epic electronic health record to analyze the prevalence, demographics, comorbid conditions and treatments for the TACs.

**Methods:**

We queried the Epic Cosmos electronic health record database for patients with diagnoses of hemicrania continua, cluster headache, paroxysmal hemicrania, and SUNCT. Prevalences, demographics were determined from this database and comorbid conditions and treatments for these conditions were analyzed.

**Results:**

Our study included 152,727 patients with cluster headache, 59,312 patients with paroxysmal hemicrania, 19,321 patients with hemicrania continua, and 6,291 patients with SUNCT. Five-year prevalence of cluster headache was highest (56.7 per 100,000), followed by paroxysmal hemicrania (22.0 per 100,000), hemicrania continua (7.2 per 100,000) and SUNCT (2.3 per 100,000). All four TACs showed a higher prevalence in women. Migraine was common in all four conditions and patients with cluster headache had the highest rates of nicotine, alcohol, and cannabis use disorders. Indomethacin was notably underutilized for the indomethacin-responsive TACs.

**Conclusion:**

We use a national electronic medical record database to give insight into elements of the TACs that have been previously limited by the relative rarity of these diseases.

**Supplementary Information:**

The online version contains supplementary material available at 10.1186/s12883-025-04314-1.

## Background

The study of populations affected by trigeminal autonomic cephalalgias (TAC) has been limited by the challenge of aggregating sufficient numbers of patients to generate full comprehension of the disease. In one of the largest studies to date of one of the TACs, patients in Norway exhibited a lifetime prevalence of cluster headache of 48.6 cases per 100,000 [1]. Beyond this, the prevalence varies significantly and few studies included sufficient numbers to generate full insight [2]. Analysis of the other TACs is more limited; the largest study of hemicrania continua (HC) involved a meta-analysis of smaller studies yielding a prevalence of 1.8% of the 9,854 patients who were evaluated for headache in a tertiary care center [3]. Paroxysmal Hemicrania (PH) is also under-evaluated; a meta-analysis suggested a pooled frequency of 0.3% of adult patients evaluated for headache in a tertiary headache center [4]. Short-lasting unilateral neuralgiform with conjunctival injection and tearing (SUNCT) was similarly found to be rare in one Norwegian register-based study, which suggested a 1-year prevalence of 1.2 cases per 100,000 [5]. 

Understanding the demographics of patients with TACs is also crucial. While previous studies suggested a male predominance of cluster headache and female predominance of the other TACs, more recent studies have called this into question and suggested comparative prevalence closer to parity in women and higher rates in women in some cases [5]. Age has also been assessed as a factor, with prevalence of cluster headache peaking between 20 and 50 [6]. Additional demographics for the TACs are limited [7]. 

Treatment of the TACs varies by type. For cluster headache, the American Headache Society assembled evidence-based guidelines for both the acute and preventive treatment of cluster headache. Subcutaneous sumatriptan, zolmitriptan nasal spray, and 100% oxygen meet criteria for “Level A” use for acute treatment while suboccipital steroid injections meet criteria for “Level A” use for preventive treatment; commonly used medications in clinic such as verapamil, lithium and melatonin only meet “Level C” use criteria [8]. Beyond these criteria, galcanezumab received FDA approval for the prevention of cluster headache [9]. 

The gold standard treatment of HC and PH is indomethacin as, by definition, an absolute response to the medication is necessary for a diagnosis [10]. Other medications have been considered for the use in HC and PH based on case reports, case series, and small retrospective studies including gabapentin, topiramate, onabotunlinumtoxinA, melatonin and COX-2 inhibitors [11, 12]. Evidence for medications for the treatment of SUNCT is limited but favors lamotrigine with other antiseizure medications and duloxetine used too [13]. 

Given the dearth of information about the demographics, comorbid conditions and treatments for the TACs, we used the Epic Cosmos electronic health record research platform to harness nationwide data from across the United States to answer these questions.

## Methods

We obtained all patient data from the Epic Cosmos research platform (Epic System Corporation, Verona, WI), an aggregated de-identified database of health systems’ electronic health records throughout the United States to be used for research and created in collaboration with a community of Epic health systems representing more than 270 million patient records from over 1,568 hospitals and 35,000 clinics from all 50 states and Lebanon [14]. Of note, the Epic Cosmos data reflects the US populations demographics [15]. Epic Cosmos is available, often without cost, to users through individual institution arrangements.

To establish prevalence, we first queried the database for patients in the United States whose chart contained an active International Classification of Disease, 10th edition diagnosis in any section during January 1, 2019 to December 31, 2023 of G44.00, G44.01, G44.02 or higher resolution for “Cluster Headache”; G44.03, G44.04 or higher resolution for “Paroxysmal Hemicrania”; G44.05 or higher resolution for “Short lasting unilateral neuralgiform headache with conjunctival injection and tearing” (SUNCT); or G44.51 for “Hemicrania Continua.” These numbers were then divided by the total queried patient population for those dates and multiplied by 100,000 to give five-year prevalence of cases per 100,000.

We then repeated these queries for the demographic groups of census region, age (both by decade and for average with standard deviation), and Race-Ethnicity. These were similarly divided by the queried patient population to give demographic specific five-year prevalence of cases per 100,000. We then queried the subgroups of patients with each TAC for the presence of ICD-10 codes for a number of comorbidities including migraine (G43.0 or higher resolution), anxiety disorder (F41 or higher resolution), depression (F32, F33 or higher resolution), irritable bowel syndrome (K58 or higher resolution), cerebrovascular disease (I60– I69 or higher resolution), and substance use disorder (F10 for alcohol related disorders, F11 for opioid related disorders, F12 for cannabis related disorders, F13 for sedative disorders, F14 for cocaine related disorders, F16 for hallucinogen related disorders, F17 for nicotine dependence, and F18 for inhalant related disorders; all higher resolution codes were also included).

We then queried the database for the active prescription of medications used for the treatment of TACs and linked to an encounter for the respective TAC including: anti-seizure medications (carbamazepine/oxcarbazepine, topiramate, lamotrigine, gabapentin, and valproic acid/valproate), anti-depressants (duloxetine/venlafaxine and nortriptyline/amitriptyline), NSAIDs (indomethacin, celecoxib, and diclofenac), steroids (methylprednisolone, prednisone, dexamethasone), triptans (sumatriptan and zolmitriptan) and others (galcanezumab, lithium, onabotulinumtoxinA, and verapamil). Of note, certain medications often associated with the treatment of the TACs could not be queried as they are either medical equipment (i.e. oxygen) or available over the counter (i.e. caffeine or melatonin). We then calculated the percentage of patients with each TAC who received each medications or any of these medications.

### Statistical analysis

Descriptive statistics were performed with Microsoft Excel including prevalence, percentage, mean, and standard deviation. No statistical tests were used in this descriptive study. Data was available only at the summary level and not at the individual patient level. Consequently, all available patients were included. This is the primary reporting of these data. In instances where Epic Cosmos returned that “10 or fewer” patients were in a specific category, this was replaced by a zero, consistent with our previous work that has used this method [16]. 

## Results

Our study included 59,312 patients with paroxysmal hemicrania, 152,727 patients with cluster headache, 19,321 patients with hemicrania continua, and 6,291 patients with SUNCT (Table 1). Five-year prevalence of cluster headache was highest (56.7 per 100,000), followed by paroxysmal hemicrania (22.0 per 100,000), hemicrania continua (7.2 per 100,000) and SUNCT (2.3 per 100,000). Prevalence varied by census region, with the Midwest having the higher prevalence of both paroxysmal hemicrania (28.7 per 100,000) and cluster headache (69.3 per 100,000), but with the Northeast having the highest prevalence of hemicrania continua (12.2. per 100,000) and SUNCT (3.1 per 100,000). The West had the lowest prevalence of all four TACs. All four TACs showed a higher prevalence in women with the highest ratio of women in hemicrania continua (2.4: 1, female to male) and lowest in cluster headache (1.3: 1, female to male). Instances of patients receiving multiple TAC diagnoses were low and this is reported in Supplementary Table 1.

**Table 1:** Prevalence and demographics for the TACs

		Paroxysmal hemicrania	Cluster headache	Hemicrania continua	SUNCT
5-year prevalence: cases per 100,000 (N, %)					
	Overall	22.0 (59312, 100%)	56.7 (152727, 100%)	7.2 (19321, 100%)	2.3 (6291, 100%)
	Midwest	28.7 (18448, 31.1%)	69.3 (44453, 29.1%)	7.3 (4703, 24.3%)	2.5 (1608, 25.5%)
	Northeast	25.2 (12459, 21.0%)	66.2 (32644, 21.3%)	12.2 (6045, 31.2%)	3.1 (1574, 25.0%)
	South	20.7 (21678, 36.5%)	55.4 (57775, 37.8%)	6.0 (6333, 32.7%)	2.2 (2302, 36.5%)
	West	14.3 (6597, 11.1%)	38.0 (17468, 11.4%)	4.7 (2188, 11.3%)	1.7 (786, 12.4%)
Prevalence by sex					
	Female	29.0 (41251, 69.5%)	60.6 (86063, 56.3%)	9.60 (13635, 70.5%)	2.84 (4041, 64.2%)
	Male	14.1 (18052, 30.4%)	52.3 (66632, 43.6%)	4.46 (5683, 29.4%)	1.76 (2250, 35.7%)
	Female to male ratio	2.3: 1	1.3: 1	2.4: 1	1.8: 1
Age					
	Average years old ± St. Dev	54 ± 20	49 ± 19	55 ± 17	55 ± 19
	Less than 5 years	0.3 (36, 0.06%)	0.4 (51, 0.03%)	NR (0, 0%)	NR (0, 0%)
	5 years − 18 years	5.62 (2006, 3.38%)	21.2 (7571, 4.95%)	0.3 (130, 0.67%)	0.4 (164, 2.60%)
	18 years − 30 years	13.6 (5516, 9.29%)	46.9 (19005, 12.4%)	2.9 (1184, 6.12%)	1.2 (506, 8.04%)
	30 years − 40 years	20.1 (7665, 12.9%)	63.7 (24240, 15.8%)	6.2 (2359, 12.2%)	1.8 (705, 11.2%)
	40 years − 50 years	27.7 (9064, 15.2%)	82.7 (27069, 17.7%)	10.5 (3455, 17.8%)	3.0 (988, 15.7%)
	50 years − 65 years	32.9 (15801, 26.6%)	84.5 (40627, 26.6%)	12.9 (6197, 32.0%)	3.7 (1805, 28.6%)
	65 years − 75 years	32.2 (9740, 16.4%)	67.9 (20483, 13.4%)	11.0 (3329, 17.2%)	3.7 (1120, 17.8%)
	75 years − 85 years	33.4 (6656, 11.2%)	52.3 (10419, 6.82%)	10.0 (2000, 10.3%)	3.6 (722, 11.4%)
	85 years or more	20.0 (2828, 4.76%)	23.0 (3262, 2.13%)	4.7 (667, 3.45%)	1.9 (276, 4.38%)
Race-ethnicity					
	White, not Hispanic or Latino	28.3 (37731, 63.6%)	73.1 (97236, 63.6%)	9.9 (13146, 68.0%)	2.92 (3884, 61.7%)
	White, Hispanic or Latino	21.3 (3620, 6.1%)	55.5 (9410, 6.1%)	7.3 (1241, 6.42%)	2.56 (435, 6.9%)
	Black or African American, not Hispanic or Latino	30.3 (10044, 16.9%)	81.2 (26853, 17.5%)	7.5 (2484, 12.8%)	3.32 (1100, 17.4%)
	Black or African American, Hispanic or Latino	28.4 (300, 0.5%)	79.8 (841, 0.5%)	9.01 (95, 0.4%)	2.84 (30, 0.4%)
	Other race, not Hispanic or Latino	33.1 (3356, 5.6%)	85.2 (8627, 5.6%)	11.2 (1135, 5.8%)	3.55 (360, 5.7%)
	Other race, Hispanic or Latino	27.7 (3265, 5.5%)	63.8 (7514, 4.9%)	8.9 (1053, 5.4%)	2.96 (349, 5.5%)
	Asian, not Hispanic or Latino	21.6 (2027, 3.4%)	41.5 (3898, 2.5%)	5.5 (519, 2.6%)	2.17 (204, 3.2%)
	Asian, Hispanic or Latino	19.9 (42, 0.0%)	54.0 (114, 0.0%)	Not reported	Not reported
	American Indian or Alaska native, not Hispanic or Latino	30.2 (412, 0.6%)	80.7 (1101, 0.7%)	9.3 (127, 0.6%)	3.74 (51, 0.8%)
	American Indian or Alaska Native, Hispanic or Latino	38.1 (214, 0.3%)	86.6 (486, 0.3%)	15.8 (89, 0.4%)	4.99 (28, 0.4%)
	Native Hawaiian or other Pacific islander, not Hispanic or Latino	22.7 (213, 0.3%)	52.9 (496, 0.3%)	4.9 (46, 0.2%)	2.34 (22, 0.3%)
	Native Hawaiian or other pacific islander, Hispanic or Latino	30.6 (71, 0.1%)	62.1 (144, 0.0%)	7.8 (18, 0.0%)	Not reported

Prevalence for all four TACs peaked in the age groups of 50–65 years but lowest prevalence in adult patients varied; prevalences for paroxysmal hemicrania and SUNCT were lowest for adult patients 18–30 years old (13.6 per 100,000 and 1.2 per 100,000, respectively) but for cluster headache and SUNCT prevalence was lowest in patients 85 or older (23.0 per 100,000 and 4.7 per 100,000, respectively). Cluster headache was noted though to have the lowest average age (49 ± 19).

Prevalence by race-ethnicity varied as well. All four TACs had the highest prevalence in American Indian or Alaska Native-Hispanic/Latino individuals. It was lowest for paroxysmal hemicrania in patients who identified as Asian-Hispanic/Latino (19.9 per 100,000), in patients who identified as Asian-Not-Hispanic/Latino for cluster headache and SUNCT (41.5 per 100,000 and 2.17 per 100,000, respectively), and in patients who identified as Native Hawaiian or Other Pacific Islande-Not-Hispanic/Latino for hemicrania continua (4.9 per 100,000).

Comorbidities were common in patients with TACs (Table 2). Notably, patients were frequently given an additional diagnosis of migraine, including 51.8% (10012/19321) of patients with hemicrania continua. Hemicrania continua patients were also most frequently diagnosed with depression (6295/19321, 32.5%), anxiety (3041/19321, 15.7%), and irritable bowel syndrome (2066/19321, 10.6%). Patients with SUNCT had the highest rate of cerebrovascular disease (653/6291, 10.4%) and those with cluster had the lowest rate (9291/152727, 6.0%). Patients with cluster headache had the highest rates of nicotine use disorder (30737/152727, 20.1%), alcohol related disorders (7208/152727, 4.7%), and cannabis related disorders (6051/152727, 3.9%). When stratified by sex, men with cluster headache had notably higher rates than women of nicotine use disorder and alcohol related disorders while women had higher rates of anxiety and depression (Supplementary Table 2).

**Table 2:** Comorbidities associated with the TACs

Comorbidity	Paroxysmal Hemicrania (*N* = 59312)	Cluster headache (*N* = 152727)	Hemicrania continua (*N* = 19321)	SUNCT (*N* = 6291)
Migraine	20,831 (35.1%)	55,639 (36.4%)	10,012 (51.8%)	2342 (37.2%)
Anxiety disorder	9148 (15.4%)	22,517 (14.7%)	3041 (15.7%)	833 (13.2%)
Depression	18,534 (31.2%)	43,742 (28.6%)	6295 (32.5%)	1844 (29.3%)
Irritable bowel syndrome	5374 (9.0%)	11,192 (7.3%)	2066 (10.6%)	485 (7.7%)
Cerebrovascular disease	5783 (9.7%)	9291 (6.0%)	1789 (9.2%)	653 (10.4%)
Alcohol related disorders	2337 (3.9%)	7208 (4.7%)	766 (3.9%)	275 (4.3%)
Opioid related disorders	2217 (3.7%)	5405 (3.5%)	693 (3.5%)	218 (3.4%)
Cannabis related disorders	1977 (3.3%)	6051 (3.9%)	568 (2.9%)	213 (3.3%)
Sedative related disorders	490 (0.8%)	1187 (0.7%)	186 (0.9%)	46 (0.7%)
Cocaine related disorders	534 (0.9%)	1476 (0.9%)	131 (0.6%)	58 (0.9%)
Hallucinogen related disorders	48 (0.0%)	114 (0.0%)	0 (0%)	0 (0%)
Nicotine dependence	9958 (16.7%)	30,737 (20.1%)	2925 (15.1%)	1027 (16.3%)
Other psychoactive substance disorders	1472 (2.5%)	30,737 (2.7%)	2925 (2.2%)	1027 (2.3%)

Prescriptions of common medications used for the treatment of TACs are included in Table 3. The vast majority of patients with a TAC received a commonly used medication including in paroxysmal hemicrania (44431/59312, 64.9%), cluster headache (115370/152727, 75.5%), hemicrania continua (16651/19321, 86.1%) and SUNCT (5044/6291, 80.1%). Steroids were broadly used in all four TACs, including paroxysmal hemicrania (27815/59312, 62.6%), cluster headache (69618/152727, 60.3%), hemicrania continua (9713/19321, 58.3%), and SUNCT (2895/6291, 57.3%). Usage of other medications varied considerably but fell into expected patterns; lamotrigine was used by 1100/6291 (21.8%) of patients with SUNCT, and verapamil in 21,860/152,727 (18.9%) of patients with cluster headache. Of the two indomethacin-responsive TACs, only hemicrania continua saw high indomethacin use (7762/19321, 46.6%), but paroxysmal hemicrania saw comparatively low utilization of the diagnostic and therapeutic medication (6533/59312, 14.7%). Both sumatriptan and zolmitriptan saw the highest rate of prescriptions in cluster headache (45112/152727, 39.1% and 4167/152727, 3.6%, respectively). Both onabotulinumtoxinA and galcanezumab were used most frequently in patients with hemicrania continua (2002/19321, 12.0% and 1874/19321, 11.2%, respectively). Of note, a very high percentage of the patients with a TAC diagnosis who received onabotulinumtoxinA also had a concurrent diagnosis of migraine but we could not delineate between these migraine diagnoses being present because the patient met International Classification of Headache Disorders, Third Edition (ICHD-3) criteria for migraine or if these diagnoses were present to facilitate insurance coverage of the medication (Supplementary Table 3).

**Table 3:** Medications prescribed for the TACs

		Paroxysmal Hemicrania (*N* = 59312)	Cluster headache (*N* = 152727)	Hemicrania continua (*N* = 19321)	SUNCT (*N* = 6291)
		N (%)	N (%)	N (%)	N (%)
At least one of the following:		44,431 (74.9%)	115,370 (75.5%)	16,651 (86.1%)	5044 (80.1%)
Anti-seizure					
	Carbamazepine/Oxcarbazepine	2426 (5.4%)	4094 (3.5%)	1469 (8.8%)	745 (14.7%)
	Topiramate	8143 (18.3%)	22,684 (19.6%)	4171 (25.0%)	1025 (20.3%)
	Lamotrigine	2299 (5.1%)	5346 (4.63%)	1142 (6.85%)	1100 (21.8%)
	Gabapentin	17,724 (39.8%)	37,264 (32.2%)	7322 (43.9%)	2351 (46.6%)
	Valproic acid/Valproate	1201 (2.7%)	2421 (2.0%)	685 (4.1%)	132 (2.6%)
Anti-depressant					
	Duloxetine/Venlafaxine	9124 (20.5%)	20,348 (17.6%)	3902 (23.4%)	990 (19.6%)
	Nortriptyline/Amitriptyline	8587 (19.3%)	20,537 (17.8%)	4156 (24.9%)	940 (18.6%)
NSAID					
	Indomethacin	6533 (14.7%)	6680 (5.79%)	7762 (46.6%)	925 (18.3%)
	Celecoxib	5212 (11.7%)	11,147 (9.6%)	2188 (13.1%)	551 (10.9%)
	Diclofenac	11,307 (25.4%)	24,911 (21.5%)	3851 (23.1%)	1202 (23.8%)
Steroids					
	Methylprednisolone/Prednisone/ Dexamethasone	27,815 (62.6%)	69,618 (60.3%)	9713 (58.3%)	2895 (57.3%)
Triptan					
	Sumatriptan	9370 (21.0%)	45,112 (39.1%)	4125 (24.7%)	996 (19.7%)
	Zolmitriptan	667 (1.5%)	4167 (3.6%)	499 (2.9%)	80 (1.5%)
Other					
	Galcanezumab	1767 (3.9%)	10,250 (8.8%)	1874 (11.2%)	312 (6.1%)
	Lithium	350 (0.78%)	1717 (1.4%)	180 (1.0%)	49 (0.9%)
	OnabotulinumtoxinA	1689 (3.8%)	4173 (3.6%)	2002 (12.0%)	266 (5.2%)
	Verapamil	3324 (7.4%)	21,860 (18.9%)	1914 (11.4%)	527 (10.4%)

## Discussion

Few large epidemiologic studies have studied paroxysmal hemicrania (PH), hemicrania continua (HC) and short-lasting unilateral neuralgiform headache attacks (SUNHA). Using nationwide electronic health record (EHR) data, our study provides new insights and adds to the existing knowledge about the trigeminal autonomic cephalalgias (TACs). This is the second study reporting higher prevalence of cluster headache (CH) in women than men. In 2008, a meta-analysis of population based epidemiological studies on cluster headache had a pooled worldwide lifetime prevalence rate of 124 per 100,000 and a 1-year prevalence of 53 per 100,000 [17]. In a nationwide study in Norway conducted between 2008 and 2016 using health records data; the prevalence of CH was 48.6 per 100,000 which is lower but somewhat comparable to the 5- year prevalence in our study of 56.7 per 100,000 [1]. 

There are two large scale epidemiological studies estimating the prevalence of paroxysmal hemicrania, hemicrania continua and SUNCT [5, 18]. The Vaga study of headache epidemiology conducted in Norway between 1995 and 1997 estimated a very high lifetime prevalence of 108 per 100,000 for “SUNCT like headache” and 54 per 100,000 for probable hemicrania continua [19]. This study utilized face to face interviews in contrast to our electronic health records-based design. In addition, an indomethacin test was not performed on patients who were given the HC diagnosis which could have led to a falsely high prevalence value. A more recent study by Hagen, using patient registry and prescription database in Norway, found 1 year prevalence rates per 100,000 of 14.6 for cluster headache, 1.2 for SUNCT/SUNA, 2.2 for hemicrania continua and 1.4 for paroxysmal hemicrania [5]. These numbers are lower than our 5-year prevalence rates per 100,000 of 56.7 for CH, 2.3 for SUNCT, 7.2 for hemicrania continua and 22 for paroxysmal hemicrania but closer than the exceedingly high figures reported in the Vaga study [5, 19]. The Hagen study and ours are both congruent with the general wisdom that cluster headaches are the most common TAC with SUNCT being the least common.

### Sex

We found a higher prevalence for all the TACs in women than men. To date, this study is the second largest epidemiological study that has found a female preponderance of cluster headache. The first was the aforementioned study by Hagen published in 2024 [5]. In that study, a female: male ratio of 1.2 was seen for cluster headache. Both studies used electronic health records; differences in seeking healthcare between genders may be a confounder that explains our similar findings with women more likely to seek healthcare [20]. Prior studies have shown a male predominance in cluster headache but notably this male predominance has been decreasing with time. In a review of 482 patients with cluster headache, the male: female ratio decreased from 6:1 for patients diagnosed in the 1960s to 2:1 for patients diagnosed in the 1990s [17, 21]. Possible explanations for the change in gender preponderance includes increased understanding of the diagnosis leading to more diagnoses in women, changes over time in the male preponderance of cigarette smoking, which is associated with cluster headache with similar rates in men and women than in the past [21–23]. Of note, when we stratified psychiatric and substance related disorders by sex, rates of cigarette smoking were higher in males such that disproportionate decreases in smoking in men could be potentially linked to lower rates of cluster headache in this cohort (Supplementary Table 2).

For paroxysmal hemicrania, retrospective surveys suggested a female predominance [24, 25]. A subsequent prospective study of 31 cases had a female: male ratio of 1:1 [26]. A recent meta-analysis based on 17 clinic-based studies and one population-based sample showed the pooled relative frequency of females with paroxysmal hemicrania was 66.7% [28]. The study by Hagen also found a female: male ratio of 4.4:1 in paroxysmal hemicrania. In our study, the female: male ratio for PH was 2.3:1 [5]. The results from our study along with these two recent published studies are the only to feature large patient numbers and add weight to earlier suggestions that paroxysmal hemicrania has a female predominance [5, 27]. 

For SUNCT/SUNA, a previous study suggested a female predominance [28]. In Hemicrania continua, the estimated female: male ratio is 2:1 [29]. Our results of a female: male ratio of 1.8:1 for SUNCT and 2.4:1 for hemicrania continua are consistent with previous studies.

### Racial/ethnic differences/geography

Most large-scale epidemiologic studies of TACs have been done in Caucasians. Hence, our study is important because it offers clues about possible racial/ethnic differences in the prevalence of these headache disorders. In our study, the prevalence for cluster headache and SUNCT was lowest in patients who identified as Asian- Not-Hispanic/Latino.

In a population-based study, one-year prevalence of cluster headache was reported as negligible in Malaysia [30]. This would appear to corroborate our findings. However, a Japanese study analyzed data from employees of large organizations who registered for health insurance and identified 21 cluster headache cases out of 21,480 participants (0.097%) which is similar to the pooled estimate in the meta-analysis by Fischera et al. of 0.12% [18, 31]. Unfortunately, a dearth of available literature exists to compare our findings to. This is an area in which more research is needed to make conclusions.

In terms of regional differences, prevalence varied by census region, with the Midwest having the highest prevalence of both paroxysmal hemicrania (28.7 per 100,000) and cluster headache (69.3 per 100,000), but with the Northeast having the highest prevalence of hemicrania continua (12.2. per 100,000) and SUNCT (3.1 per 100,000). The West had the lowest prevalence of all four TACs.

Cluster headache is known to show a seasonal predilection. A case-crossover study which studied the association between cluster and weather changes showed that seasonal changes from winter to spring and from autumn to winter increased the frequency of cluster periods [32]. Changes in temperature following preceding warm or cold periods were also associated with the occurrence of cluster periods, suggesting that warm or cold periods may have a priming effect on cluster periods. It could be inferred from this that regions with more temperature variations may have more cluster headache bouts/periods. The West census region has less temperature variation across the year, and this may account for the lower prevalence of cluster headache.

### Comorbidities/Indomethacin underutilization

Patients in the study were frequently given an additional diagnosis of migraine. 35.1%, 36.4%, 37.2% and 51.8% in paroxysmal hemicrania, cluster headache, SUNCT and hemicrania continua respectively. Having comorbid migraine may increase the likelihood of receiving a diagnosis of a TAC given that the patient is more likely to be under the care of a neurologist or headache specialist. With regards to 51.8% of patients with hemicrania continua having comorbid migraine; migrainous features including photophobia and phonophobia are commonly present in patients with hemicrania continua and strictly unilateral migraines occur in some patients hence misdiagnosis could be a factor at play [33]. Additionally, given the high rates of migraine diagnoses in patients who received a TAC diagnosis and treatment with onabotulinumtoxinA, the migraine code may also be present to facilitate coverage of treatment; 94.4% of patients with hemicrania continua who received onabotulinumtoxinA also received a diagnosis of migraine.

Depression and anxiety are well established as comorbidities in primary headache disorders and cluster headache has been linked to increased suicidal ideation [34–36]. In addition, cigarette smoking is a known risk factor in cluster headache with as many as 88% of patients in observational reports and surveys reported to be chronic cigarette smokers [37, 38]. 

With regards to prescriptions, the most notable observation in our study was the lower-than-expected use of indomethacin in patients with a hemicrania continua and paroxysmal hemicrania diagnosis. Given that these disorders are defined by an essential response to indomethacin, one would expect more associated prescriptions. Indomethacin is associated with gastrointestinal side effects; hence it is possible that a presumptive diagnosis of hemicrania continua was made but other better tolerated, albeit with less efficacy data, medication options were utilized. It does, however, highlight the main limitation of our study; diagnosis of the TACs were based on patients receiving those diagnostic codes and it is possible that those patients did not meet full ICHD-3 criteria for the respective TAC, including a complete response to indomethacin as is required for the diagnosis of paroxysmal hemicrania or hemicrania continua, or were incorrectly diagnosed and had another primary headache disorder such as migraine.

Our study has a few limitations as a result of using the Epic Cosmos platform. Since the Epic Cosmos research platform relies on data from Epic itself, there is the possibility of selection bias from patients whose treating institution used Epic and not other EHRs. Additionally, diagnosis of the TACs were based on patients receiving those diagnostic codes and it is possible that those patients did not meet full ICHD-3 criteria for the respective TAC or were incorrectly diagnosed and actually had another primary headache disorder such as migraine. Moreover, that some patients received diagnoses of two TACs also suggests a degree of diagnostic uncertainty inherent in using diagnostic codes for identification of patients. Medication information as well is potentially incomplete as Epic may not capture all indomethacin prescriptions, such as those that were tried prior to establishing with the current documenting physician, potentially explaining the lower-than-expected indomethacin prescriptions to some extent. Similarly, extension of these results beyond the United States may be difficult due to difference in demographics and practice patterns.

## Conclusion

Our study is among the largest to date of the TACs and notably shows a female predominance of all TACs as well as lowest prevalence among Asian and Hispanic/Latino patients as well as regional variance in prevalence. The data confirmed the association with known co-morbidities such as migraine, depression, and anxiety, but also revealed insight into lesser explored co-morbidities such as cerebrovascular disease and cannabis-related disorders. Critically, indomethacin is underutilized in the treatment of hemicrania continua and paroxysmal hemicrania and increased awareness of this as a treatment, as well as additional options for those who cannot tolerate indomethacin are required.

## Supplementary Information

Supplementary Material 1.

Supplementary Material 2.

Supplementary Material 3.

## Data Availability

All data were obtained through the Epic Cosmos Research Platform available at https://cosmos.epic.com/.
