# Supplementary material for: Prevalence, demographics, comorbidities, and treatment patterns of patients with the trigeminal autonomic cephalalgias: a retrospective analysis of United States electronic health records
Source: BMC Neurol. 2025 Jul 21;25:299. doi: 10.1186/s12883-025-04314-1 (PMC12278493; doi:10.1186/s12883-025-04314-1)
Supplement: Supplementary file 1 — Supplementary Material 1. [file 12883_2025_4314_MOESM1_ESM.docx]

Supplementary Table 1. Co-diagnoses of TACs

|  | Number | Cluster Headache | Hemicrania continua | Paroxysmal Hemicrania | SUNCT |  |
| --- | --- | --- | --- | --- | --- | --- |
| Cluster Headache | 152727 |  | 0.9% | 0.9% | 0.4% | 2.2% |
| Hemicrania Continua | 19321 | 7.0% |  | 4.4% | 0.4% | 11.8% |
| Paroxysmal Hemicrania | 59312 | 2.5% | 1.6% |  | 0.3% | 4.4% |
| SUNCT/SUNHA | 6291 | 9.2% | 1.3% | 2.8% |  | 13.4% |
