# Supplementary material for: Prevalence, demographics, comorbidities, and treatment patterns of patients with the trigeminal autonomic cephalalgias: a retrospective analysis of United States electronic health records
Source: BMC Neurol. 2025 Jul 21;25:299. doi: 10.1186/s12883-025-04314-1 (PMC12278493; doi:10.1186/s12883-025-04314-1)
Supplement: Supplementary file 2 — Supplementary Material 2. [file 12883_2025_4314_MOESM2_ESM.docx]

Supplementary Table 2. Patients with a TAC who received onabotulinumtoxinA by migraine diagnosis

|  |  | Paroxysmal Hemicrania | Cluster Headache | Hemicrania continua | SUNCT |
| --- | --- | --- | --- | --- | --- |
| Total Patients | | 59312 | 152727 | 19321 | 6291 |
| Total Patients who received onabotulinumtoxinA | | 1841 | 4528 | 2039 | 278 |
|  | With a migraine diagnosis | 86.4% | 90.8% | 94.4% | 89.2% |
|  | With no migraine diagnosis | 13.6% | 9.2% | 5.6% | 10.8% |
