# Supplementary material for: Prevalence, demographics, comorbidities, and treatment patterns of patients with the trigeminal autonomic cephalalgias: a retrospective analysis of United States electronic health records
Source: BMC Neurol. 2025 Jul 21;25:299. doi: 10.1186/s12883-025-04314-1 (PMC12278493; doi:10.1186/s12883-025-04314-1)
Supplement: Supplementary file 3 — Supplementary Material 3. [file 12883_2025_4314_MOESM3_ESM.docx]

Supplementary Table 2. Psychiatric and substance use disorder comorbidities by sex

|  |  | Paroxysmal Hemicrania | | Cluster Headache | | Hemicrania continua | | SUNCT | |
| --- | --- | --- | --- | --- | --- | --- | --- | --- | --- |
|  |  | Female | Male | Female | Male | Female | Male | Female | Male |
| Number |  | 41251 | 18052 | 86063 | 66632 | 1365 | 5683 | 4041 | 2250 |
| Diagnosis Percentage |  |  |  |  |  |  |  |  |  |
|  | Anxiety | 17.8% | 11.0% | 18.8% | 10.6% | 17.3% | 11.4% | 15.5% | 10.0% |
|  | Depression | 34.8% | 23.1% | 35.2% | 20.6% | 35.5% | 24.5% | 33.1% | 22.5% |
|  | Alcohol Use Disorder | 3.0% | 7.3% | 3.2% | 7.2% | 2.9% | 6.7% | 3.2% | 6.9% |
|  | Opioid Use Disorder | 3.7% | 3.9% | 3.4% | 3.7% | 3.4% | 4.3% | 3.2% | 3.5% |
|  | Cannabis Use Disorder | 3.1% | 4.6% | 3.5% | 4.9% | 2.6% | 4.0% | 3.1% | 4.3% |
|  | Sedative Use Disorder | 0.9% | 0.7% | 0.9% | 0.7% | 1.0% | 0.9% | 0.7% | 0.6% |
|  | Cocaine Use Disorder | 0.6% | 1.2% | 0.6% | 1.3% | 0.5% | 1.1% | 0.5% | 1.5% |
|  | Hallucinogen Use Disorder | 0.0% | 0.1% | 0.0% | 0.1% | 0.0% | 0.0% | 0.0% | 0.0% |
|  | Other Stimulant Disorder | 0.8% | 1.3% | 0.8% | 1.3% | 0.6% | 1.2% | 0.4% | 1.1% |
|  | Nicotine Use Disorder | 14.8% | 20.2% | 16.0% | 24.8% | 13.1% | 19.2% | 14.1% | 19.4% |
|  | Other Psychoactive Use Disorder | 2.0% | 3.1% | 2.2% | 3.2% | 1.9% | 2.6% | 1.8% | 2.6% |
